# Knowledge, opinions and expectations of adults concerning personalised genotype-based dietary recommendations: a German survey

**DOI:** 10.1017/S1368980020004152

**Published:** 2021-05

**Authors:** Sandra Bayer, Theresa Drabsch, Gunther Schauberger, Hans Hauner, Christina Holzapfel

**Affiliations:** 1 Institute for Nutritional Medicine, School of Medicine, Technical University of Munich, Munich 80992, Germany; 2 Chair of Epidemiology, Department of Sport and Health Sciences, Technical University of Munich, Munich, Germany; 3 ZIEL – Institute for Food & Health, Technical University of Munich, Freising, Germany

**Keywords:** Personalised nutrition, Genotype-based nutrition, Survey, German

## Abstract

**Objective:**

To assess the knowledge, opinions and expectations of persons with and without obesity concerning personalised genotype-based nutrition.

**Design:**

Questions about nutrition, weight management and personalised genotype-based dietary recommendations were asked via standardised telephone-based interviews. Sociodemographic and anthropometric data were collected. The data were statistically weighted by age, gender, education, domicile and BMI.

**Setting:**

Germany.

**Participants:**

Representative sample of the German population (*n* 1003) randomly sampled via a scientific Random Digit dial method plus 354 adults with a BMI ≥ 30·0 kg/m^2^ to enlarge the sample.

**Results:**

Data of 1357 participants were analysed (51·1 % female, age: 50·5 ± 18·5 years, 15·9 % adults with a BMI ≥ 30·0 kg/m^2^). About 42 % or 19 % of the survey participants stated to know the terms personalised dietary recommendation or genotype-based dietary recommendation, respectively. Of those, 15·8 % indicated to have an experience with a personalised or genotype-based dietary recommendation. Almost 70 % of the survey participants believed that a genotype-based dietary recommendation is a reasonable measure for weight management. About 55 % of the survey participants pointed out that a genotype-based dietary recommendation is an effective concept in general. One-third of the survey participants (34·6 %) indicated to conceive the usage of a genotype-based dietary recommendation.

**Conclusion:**

Most of the survey participants did not know the term personalised or genotype-based dietary recommendation. One-third of the study participants are interested to use a genotype-based dietary recommendation. Therefore, more education of the public is necessary to properly help people making informed and serious decisions and assessing commercially available direct-to-consumer genetic tests.

Individual nutritional needs as well as scientific evidence indicate that dietary recommendations according to the ‘one size fits all’-principle are no longer useful or acceptable. Therefore, the research efforts on the development and promotion of healthy eating advice tailored to the nutritional needs of an individual increased in the last years. Up to now, there is no consistent definition of the term personalised nutrition. In fact, personalised dietary recommendations can be based on different characteristics of a person, among others, phenotype (e.g. body weight), metabolism (e.g. glucose), microbiome, or genetics^(1)^. Stewart-Knox *et al.* defined personalised nutrition as a ‘healthy eating advice that is tailored to suit an individual based on their own personal health status, lifestyle and/or genetics’^(2)^.

One approach is to make personalised dietary recommendations based on the genetic background of a person^(1)^. Approximately 941 gene loci were found to be associated with the BMI, which account for approximately 6 % of BMI variation^(3)^. Moreover, several SNP have been identified to be associated with the body weight^(4,5,6,7,8)^, whereas the effect of each single SNP is rather small. These associations are mostly based on epidemiological data, which lack to proof causality. Due to the small effect size, the clinical relevance is questionable. Moreover, the function of most of the SNP identified in genome-wide association studies is unknown. However, the A allele of SNP rs571312 of the melanocortin-4 receptor (*MC4R*) gene is associated with an increased BMI by 0·23 kg/m^2(7)^. Furthermore, homozygous carriers of the A allele of SNP rs9939609 of the fat mass and obesity-associated (*FTO*) gene weigh up to three kilogrammes more than non-risk allele carriers^(4)^. The investigation of the cumulative effect of twelve SNP in 20 431 individuals from the population-based European Prospective Investigation into Cancer and Nutrition (EPIC)–Norfolk cohort has shown that all SNP combined explained 0·9 % of BMI variation^(9)^. Although the reported associations of this sort open a promising approach for the treatment of overweight and obesity, there is little scientific evidence on genotype-based dietary recommendations for weight loss. For instance, the Food4Me project investigated different levels of tailored nutrition. In comparison to the control group, a personalised dietary recommendation independent of the level of personalisation led to a significantly greater weight loss^(10)^, whereas the personalisation based on specific SNP had no further benefit in this study compared to other strategies of personalisation^(10)^. There is also lack of evidence that obesity-related genetic loci are associated with nutrition^(11,2,13)^. One systematic review has shown that there is no evidence for an association between different SNP and energy or macronutrient intake^(11)^. In another systematic review, each copy of the *FTO* risk allele was significantly associated with lower total energy intake of 6·46 kilocalories per day. Total fat and protein intake were higher in individuals carrying the *FTO* risk allele^(12)^. Furthermore, there was no evidence for an association between *FTO* SNP and the amount of weight loss^(13)^.

However, commercially available direct-to-consumer (DTC) genetic tests focusing on dietary recommendations based on SNP are on the market. Although there is no scientific evidence for genotype-based dietary recommendations, providers of DTC genetic testing often claim on their homepages to increase the persons’ well-being and to support weight loss. Thus, due to the lack of scientific evidence^(14)^ and the missing control of test results by healthcare professionals, the international professional societies advise the general population as well as medical doctors and nutritional experts against the usage of DTC genetic testing for genotype-based dietary recommendations^(15,16,17)^.

As most experts are aware of the technical details of genotype-based dietary recommendations, they might be able to evaluate the commercial market. But this might not be the case for patients or the general, lay population. It is questionable whether the general population can make an ethically responsible decision to DTC tests. Therefore, this representative as well as target group-specific survey aimed to identify the knowledge, opinions and expectations of adults concerning personalised genotype-based dietary recommendations. Against the background that there is no clear evidence about the benefit of genotype-based dietary recommendations and that the market of commercially available DTC genetic testing is increasing, it is an effort to identify the knowledge base in the target group of DTC tests. The results might open research questions for future studies and might improve the approaches of personalised dietary recommendations based on consumer’s perceptions and needs.

## Methods

### Sample

This German telephone-based survey was conducted between January and March 2019 by USUMA GmbH, a social research and market analysis company. Landline or mobile phone users being 18 years of age and older were randomly sampled via a scientific Random Digit Dialing (RDD) method (ADM-Sampling System, Dual Frame). The used RDD sampling frame followed a further developed version of the Gabler–Häder procedure, which allowed potential participants who were not listed in official registers to be contacted. As 15 % of the German population does not have access to landline connections, about 40 % of the initial sample were mobile numbers to cover so-called ‘mobile-only households’. The Kish selection grid was used to randomly select a subject within one household.

For the intended representative sample, 2361 subjects were invited. From these, 42·5 % (*n* 1003) participated in the survey, which is comparable to other telephone-based surveys in Germany^(18,19)^. Additional target group-specific interviews were conducted to reach 500 subjects with obesity (BMI ≥ 30·0 kg/m^2^) to compare the results between persons with a BMI < 30·0 kg/m^2^ and a BMI ≥ 30·0 kg/m^2^. These people were included through the same sampling method by making use of a short screening process at the beginning of the interview. In the end, data from 1357 persons were available, out of whom 505 persons had a BMI ≥ 30·0 kg/m^2^. The interview was about 25 min long and was conducted by trained staff.

### Questionnaire

The survey team developed a standardised survey-specific questionnaire. In advance, one focus group with persons with a BMI < 30·0 kg/m^2^ and one focus group with persons with a BMI ≥ 30·0 kg/m^2^ have been conducted to brainstorm the topic personalised dietary recommendations to develop an understandable questionnaire in plain language. The first draft of the questionnaire was pretested in paper form. The pre-final version of the questionnaire was evaluated in terms of understanding and length by computer-assisted telephone-based interviews.

The final version of the questionnaire started with a short introduction including information about the research project, voluntary participation and data protection. Furthermore, sociodemographic data (age, gender, marital status, education, occupation and migration) were collected (10 questions). Self-reported anthropometric data (height and weight) were used for BMI calculation according to the WHO criteria^(20)^ (four questions). As the BMI was a major parameter for the subanalyses, a computer-based method was implemented. This method allowed us to calculate the BMI without naming the actual weight. First, body height, which was mandatory for participation, was asked. By reluctance to name the body height, the person’s participation was interrupted and disregarded. After mentioning body height, the participant was asked for his body weight to calculate the BMI with the equation BMI = weight in kilogram/(height in metres)^2^. In case of reluctance to report the body weight, the interviewer asked for pre-calculated weight ranges that allowed to calculate the participant’s BMI according to the WHO criteria (underweight/normal weight: BMI < 25·0 kg/m^2^, overweight: BMI 25·0-29·9 kg/m^2^, obesity: BMI ≥ 30·0 kg/m^2^). This method was applied for five participants who did not report their body weight. The 42-item questionnaire included further questions on four main topics – weight management (four questions), questions on nutrition (four questions), state of knowledge of personalised genotype-based dietary recommendations (six questions) and opinions and expectations of personalised genotype-based dietary recommendations (fourteen questions). After questions about the participant’s state of knowledge on personalised genotype-based dietary recommendations were asked, the following definition of genotype-based dietary recommendation was given to guarantee that every participant had the same understanding: ‘A genotype-based dietary recommendation is a dietary recommendation which is adapted to each individual. In addition to lifestyle, it is especially based on the genetic information of the individual who is receiving the dietary recommendation. Therefore, it is not about genetically engineered food but rather a dietary recommendation considering the individual’s genetic background’.

Screening questions were applied for individualising the questionnaire which means that some questions were skipped when the participant answered a former question with, for example, ‘no’ or ‘I do not know’. The questionnaire consisted of open-ended, semi-closed and closed questions with single- and multi-choice answer options. In addition, Likert scales were used to range the importance of the different variables within one question. To avoid influencing the subject’s answer, most of the questions have ‘no answer’ options.

### Data analysis

Before the analysis, data were statistically weighted by age, gender, education and domicile according to the data from the Federal Statistical Office for gaining representative results for the German population by iterative proportional fitting^(21)^. According to the weighted representative sample of adults in Germany (1003 interviews), 15·9% of them had a BMI ≥ 30·0 kg/m^2^. Based on this percentage, the entire sample including the top-up sample was proportionalised. Due to this weighing of data, 852 interviewed subjects with a BMI < 30·0 kg/m^2^ represent 1141 (84·1 %) persons and 505 interviewed subjects with a BMI ≥ 30·0 kg/m^2^ represent 216 (15·9 %) persons within the total sample (1357 interviews). Thereafter, the percental distribution of the BMI categories was according to the population demographics and no further iterative proportional fitting was done. In the interview, the different variables within each question were randomly chosen for each survey participant to avoid order bias. The number of participants varied per question due to screening questions, which means that only persons who give a certain answer to a further question got the following question (e.g. persons who answered ‘no’ to the question ‘Could you generally conceive to make use of genotype-based dietary recommendation as a service?’ did not receive the question ‘By which institution would you make use of a genotype-based dietary recommendation?’). This information is indicated in the respective tables. Furthermore, answers with ‘no answer’ or ‘do not know’ were not included in statistical analysis to present clear and understandable results. The present analysis is focused on dietary aspects; therefore, 27 questions were used for statistical analysis. The results of the other questions are published elsewhere^(22)^. The descriptive statistical analysis (frequencies and percentages) was done with SPSS statistical software (SPSS version 25·0, SPSS Inc., Chicago, IL, USA). All analyses were performed for the total sample as well as for subgroups, such as BMI (BMI < 30·0 kg/m^2^, BMI ≥ 30·0 kg/m^2^), gender, age and education, because subgroup differences were assumed. Furthermore, in the statistical software R^(23)^, weighted chi-squared independence tests were performed to compare the respective subgroups. For each test, the corresponding *P*-values for the null hypothesis of equal proportions among all subgroups were reported. The *P*-values have been corrected according to Holm’s correction^(24)^ by considering all 216 hypotheses from this manuscript. A significance level of 0·05 has been considered as statistically significant. Data are shown as numbers and percentages of the total sample.

## Results

### Characteristics of the survey participants and subjects

The survey participants (*n* 1357) consisted of 51·1 % (694/1357) women. Considering the BMI, 15·9 % (216/1357) of the survey participants had a BMI ≥ 30·0 kg/m^2^ (Table 1). The mean age was 50·5 ± 18·5 years. Half of the participants were married (53·0 %, 718/1353) and 37·7 % (482/1279) were educated over 12 or 13 years. Furthermore, 53·6 % (726/1356) of the participants were employed and 18·5 % (243/1311) had a migration background (Table 1).

**Table 1:** Characteristics of the survey participants and subjects

Variable	Number	
	*n*/N†	%
Gender		
Female	694/1357	51·1
Male	663/1357	48·9
Age (years)		
18–35	345/1357	25·5
36–65	690/1357	50·8
> 65	322/1357	23·7
BMI (kg/m2)		
< 18·5	19/1357	1·4
18·5–24·9	662/1357	48·8
25–29·9	459/1357	33·8
≥ 30	216/1357	15·9
Marital status		
Single	415/1353	30·7
Married	718/1353	53·0
Divorced/widowed	220/1353	16·3
Education*		
Student	3/1279	0·2
8/9 years	360/1279	28·1
10 years	424/1279	33·2
12/13 years	482/1279	37·7
No education	10/1279	0·8
Occupation	726/1356	53·6
Immigrant background	243/1311	18·5

Data are statistically weighted by age, gender, education, domicile and body mass index (BMI).

* What is your highest level of education? Possible answers: still studying, certificate of secondary education (8/9 years), a general certificate of secondary education (10 years), higher education entrance certification (12/13 years), no student/education, no answer.

† Persons with answers ‘no answer’ are not included in statistical analysis. The number of those answers can be calculated by the difference between the total sample (*n* 1357) and the number of answers given for each variable.

### Weight history

Almost 60 % (58·2 %, 790/1356) of the survey participants were satisfied with their body weight. However, this percentage is threefold higher in the subgroup of persons with a BMI < 30·0 kg/m^2^ compared to people with a BMI ≥ 30·0 kg/m^2^ (65·4 % *v*. 20·3 %, *P* = 9e–33) (Table 2). More than half (54·9 %, 745/1357) of the participants ever tried to lose 3 kg of their body weight with a statistically significant difference in the subgroup of persons with a BMI ≥ 30·0 kg/m^2^ compared to persons with a BMI < 30·0 kg/m^2^ (85·7 % *v*. 49·0 %, *P* = 5e–21) (Table 2). Women have tried more often to lose weight than men (60·0 % *v*. 49·5 %, *P* = 2e–02) (Table 2).

**Table 2:** Weight history and knowledge and opinions on a genotype-based dietary recommendation

Variable	Total		BMI (kg/m^2^)			Gender			Age (years)				Education (years)				
			< 30	≥ 30		Women	Men		18–35	36–65	> 65		Student/none	8/9	10	12/13	
	n/N¶¶	%	%	%	*P*	%	%	*P*	%	%	%	*P*	%	%	%	%	*P*
**Weight**																	
Satisfaction*	790/1356	58·2	65·4	20·3	9e–33	56·2	60·3	1e–0	63·3	57·1	55·0	1e–0	93·6	47·8	56·4	63·7	4e–04
Weight reduction†	745/1357	54·9	49·0	85·7	5e–21	60·0	49·5	2e–02	47·8	59·0	53·6	4e–01	53·2	56·0	55·7	55·5	1e–0
**Personalised genotype-based diet**																	
Knowledge of personalised dietary recommendation‡,***	568/1357	41·8	40·5	48·8	1e–0	43·1	40·6	1e–0	49·8	42·5	31·9	3e–03	0·0	35·4	38·5	51·2	2e–05
Knowledge of genotype-based dietary recommendation§,***	262/1357	19·3	19·0	20·7	1e–0	20·7	17·8	1e–0	22·6	18·7	17·2	1e–0	8·4	18·7	20·2	21·3	1e–0
Different kinds of nutritional recommendation‖,***	798/1113	71·5	71·0	74·3	1e–0	73·2	69·9	1e–0	72·9	73·7	64·5	1e–0	21·0	72·3	71·9	72·8	1e–0
Experience of personalised or genotype-based dietary recommendation¶,***	101/635†††	15·7	14·4	22·1	1e–0	13·5	18·9	1e–0	13·1	18·2	13·7	1e–0	0·0	21·6	13·3	16·3	1e–0
Expectation of genotype-based dietary recommendation**,***																	
Health maintenance	1146/1324	86·6	85·9	90·0	1e–0	87·8	85·3	1e–0	95·6	87·5	74·1	8e–12	100·0	83·4	88·5	88·4	1e–0
Weight management	1014/1257	80·6	79·7	85·5	1e–0	82·8	78·7	1e–0	86·4	83·0	68·3	5e–06	100·0	76·1	81·4	85·9	2e–01
Wellbeing/fitness	1137/1333	85·2	84·8	87·7	1e–0	88·2	82·4	5e–01	87·5	85·7	81·6	1e–0	79·9	83·9	85·3	87·6	1e–0
Longer lifetime	1016/1286	79·0	78·2	83·4	1e–0	78·1	80·0	1e–0	80·3	81·2	71·9	7e–01	63·6	78·1	79·6	80·9	1e–0
Healing of illness	1048/1305	80·3	80·3	80·6	1e–0	84·1	76·5	1e–01	83·1	81·2	74·8	1e–0	79·9	79·1	81·4	80·4	1e–0
Relief of discomfort	1133/1307	86·7	86·6	87·0	1e–0	89·8	83·3	9e–02	90·6	87·0	80·7	3e–01	100·0	84·4	88·7	87·8	1e–0
Meaningfulness of genotype-based dietary recommendation††,‡‡‡																	
By serious sport	811/1296	62·6	61·9	66·5	1e–0	64·6	60·5	1e–0	67·6	62·3	57·3	1e–0	92·8	62·2	62·6	61·8	1e–0
By food intolerance	992/1308	75·8	75·6	77·0	1e–0	79·4	72·2	4e–01	71·2	79·4	73·0	1e–0	72·2	77·3	69·4	79·3	7e–01
For wellbeing	896/1323	67·7	66·3	75·2	1e–0	69·4	66·2	1e–0	64·2	67·8	72·1	1e–0	59·7	77·7	65·1	63·1	7e–03
For maintaining health	1022/1325	77·1	75·6	85·4	3e–01	80·9	73·0	2e–01	77·6	76·0	79·0	1e–0	65·6	85·3	73·9	73·7	2e–02
By diabetes	1031/1297	79·5	78·5	84·9	1e–0	80·9	78·0	1e–0	79·5	80·9	75·5	1e–0	85·8	80·0	76·8	81·3	1e–0
By weight problems	941/1319	71·3	69·9	79·6	8e–01	73·8	69·1	1e–0	74·7	70·4	69·7	1e–0	30·7	77·4	68·1	72·6	3e–02
By cancer	777/1266	61·4	61·0	63·8	1e–0	64·0	58·8	1e–0	54·8	64·0	63·3	1e–0	72·7	67·6	57·8	58·3	1e–0
By high blood lipids	959/1299	73·8	72·4	81·3	1e–0	79·4	68·2	9e–04	71·8	74·1	75·7	1e–0	48·5	74·9	71·3	75·7	1e–0
By CVD	943/1310	72·0	70·2	81·6	1e–01	77·5	66·0	8e–04	72·8	72·2	70·1	1e–0	37·4	73·6	69·7	73·1	1e–0
Usage of genotype-based dietary recommendation‡‡,‡‡‡	414/1198	34·6	31·7	49·2	6e–04	38·6	30·2	4e–01	38·8	38·5	21·7	2e–04	18·2	32·1	29·7	39·8	1e–0
Efficiency of genotype-based dietary recommendation§§,‡‡‡	631/1140	55·3	54·7	59·0	1e–0	60·1	50·4	3e–01	61·1	57·5	43·4	1e–02	33·3	58·4	51·5	62·3	1e–0
Establishment of genotype-based dietary Recommendation‖‖,‡‡‡	398/1039	38·3	36·6	47·2	1e–0	43·0	33·9	6e–01	39·2	42·2	27·5	2e–01	37·8	42·6	36·8	41·0	1e–0

BMI, body mass index; *P*, *P*-value.

Data are statistically weighted by age, gender, education, domicile and BMI.

* How satisfied are you with your body weight? Possible answers from 1 = very dissatisfied, 2, 3, 4 to 5 = very satisfied, no answer; shown is ‘4’ and ‘5’ as answer.

† Have you ever tried to lose 3 kg of your body weight? Possible answers: yes, no, no answer; shown is ‘yes’ as answer.

‡ Do you know the term ‘personalised dietary recommendation’ as a counselling or nutritional tip? Possible answers: yes, no, no answer; shown is ‘yes’ as answer.

§ Do you know the term ‘genotype-based dietary recommendation’ as a counselling or nutritional tip? Possible answers: yes, no, no answer; shown is ‘yes’ as answer.

‖ Do you think that personalised and genotype-based dietary recommendations are different kinds of nutritional recommendation? Possible answers: yes, no, do not know, no answer; shown is ‘yes’ as answer.

¶ Have you already experienced personalised or genotype-based dietary recommendation? Possible answers: yes, no, no answer; shown is ‘yes’ as answer.

** Which effect would you expect of a genotype-based dietary recommendation on health? Possible answers from 1 = no expectation, 2 = (rather) minor expectation, 3 = (rather) high expectation; do not know, no answer; shown is ‘2’ and ‘3’ as answer.

†† In which of the following situation would you consider a genotype-based dietary recommendation as meaningful? Possible answers: yes, no, no answer; shown is ‘yes’ as answer.

‡‡ Could you generally conceive to make use of genotype-based dietary recommendation as a service? Possible answers: yes, no, no answer; shown is ‘yes’ as answer.

§§ Do you believe genotype-based dietary recommendation to be an effective concept? Possible answers: yes, no, do not know, no answer; shown is ‘yes’ as answer.

‖‖ Do you think genotype-based dietary recommendations will establish itself in the future? Possible answers: yes, no, do not know, no answer; shown is ‘yes’ as answer.

¶¶ Persons with answers ‘no answer’ or ‘do not know’ are not included in statistical analysis. The number of those answers can be calculated by the difference between the total sample (*n* 1357) and the number of answers given for each variable.

*** Before the definition of a genotype-based dietary recommendation was given.

††† Screening question, which means that only persons who give certain answer to further item got this question.

‡‡‡ After the definition of a genotype-based dietary recommendation was given.

### Knowledge and opinions

When asked for their knowledge and opinions concerning personalised genotype-based dietary recommendations, 41·8 % (568/1357) stated to know the term personalised dietary recommendation, while 19·3 % (262/1357) stated to know the term genotype-based dietary recommendation (Table 2). The indication of knowing the term of a personalised dietary recommendation differed statistically significant between age groups (18–35 years: 49·8 % *v*. 36–65 years: 42·5 % *v*. older than 65 years: 31·9 %, *P* = 3e–03). Comparing education subgroups also statistically significant results have been observed (student/none: 0·0 % *v*. 8/9 years: 35·4 % *v*. 10 years: 38·5 % *v*. 12/13 years: 51·2 %, *P* = 2e-05) (Table 2). Over 70 % (71·5 %, 798/1113) of the survey participants thought that personalised and genotype-based dietary recommendations are two different kinds of recommendations. Moreover, 15·7 % (101/637) of the survey participants indicated to have already experienced a genotype-based dietary recommendation (Table 2). Concerning these questions, no statistically significant differences among the subgroups were found (Table 2).

### Expectations and willingness

The majority of the survey participants expected a health benefit of a genotype-based dietary recommendation (Table 2). Moreover, most of the survey participants considered a genotype-based dietary recommendation meaningful in different health situations. Almost 35 % (34·6 %, 414/1198) of the survey participants could generally conceive to make use of a genotype-based dietary recommendation with statistically significant differences between the BMI subgroups (31·7 % *v*. 49·2 %, *P* = 6e–04) and the age subgroups (18–35 years: 38·8 % *v*. 36–65 years: 38·5 % *v*. older than 65 years: 21·7 %, *P* = 2e–04). Over half (55·3 %, 631/1140) of the study participants believed that a genotype-based dietary recommendation is an effective concept in general (Table 2). The results concerning this question differed statistically significant among the age subgroups (18–35 years: 61·1 % *v*. 36–65 years: 57·5 % *v*. older than 65 years: 43·4 %, *P* = 1e–02) (Table 2). Almost all participants would provide information about different kinds of personal data to receive a genotype-based dietary recommendation (Table 3). However, half of the participants would provide data about their whereabouts (49·5 %, 204/413) with statistically significant differences between the age subgroups (18–35 years: 29·7 % *v*. 36–65 years: 54·8 % *v*. older than 65 years: 66·7 %, *P* = 2e–05) (Table 3).

**Table 3:** Expectation and willingness concerning a genotype-based dietary recommendation (*n* 414)

Variable	Total		BMI (kg/m2)			Gender			Age (years)				Education (years)				
			< 30	≥ 30		Women	Men	*P*	18–35	36–65	> 65	*P*	Student/none	8/9	10	12/13	*P*
	*n*/N‡	%	%	%	*P*	%	%		%	%	%		%	%	%	%	
Providing personal data for genotype-based dietary recommendation*,§,‖																	
Age	381/413	92·3	91·0	97·2	1e–0	90·3	94·7	1e–0	93·4	92·6	89·4	1e–0	100·0	95·9	94·5	93·4	1e–0
Gender	383/414	92·5	91·7	95·1	1e–0	92·5	92·5	1e–0	89·4	94·4	91·5	1e–0	100·0	91·6	95·9	93·6	1e–0
Whereabouts (GPS, geo data)	204/413	49·5	47·2	57·4	1e–0	43·3	57·3	6e–01	29·7	54·8	66·7	2e–05	100·0	55·1	57·3	43·7	1e–0
Height	384/414	92·9	91·5	97·5	1e–0	94·0	91·4	1e–0	87·5	94·7	96·3	1e–0	100·0	94·2	94·5	93·9	1e–0
Body weight	387/414	93·6	92·3	98·0	1e–0	94·3	92·6	1e–0	88·5	94·9	98·4	1e–0	100·0	89·8	94·2	96·4	1e–0
Health status	374/412	90·9	90·3	92·9	1e–0	91·1	89·8	1e–0	96·5	87·8	90·0	1e–0	100·0	90·2	88·2	94·2	1e–0
Eating behaviour	379/413	91·9	90·4	96·9	1e–0	89·6	93·4	1e–0	92·9	92·1	89·7	1e–0	100·0	94·5	94·1	93·1	1e–0
Physical activity	388/414	93·8	92·8	97·1	1e–0	93·2	94·5	1e–0	92·2	93·7	97·1	1e–0	100·0	96·1	96·8	92·7	1e–0
Sleeping period	367/410	89·5	89·1	90·9	1e–0	88·9	90·3	1e–0	84·1	91·2	93·4	1e–0	100·0	92·0	90·8	85·1	1e–0
Blood sample	317/412	76·9	74·4	85·6	1e–0	72·9	82·0	1e–0	82·7	75·4	72·1	1e–0	100·0	77·1	74·4	80·5	1e–0
Genetic make-up/genetic material (e. g. saliva sample)	299/409	73·2	70·5	82·0	1e–0	74·5	71·3	1e–0	72·3	73·1	74·2	1e–0	100·0	80·2	71·6	75·0	1e–0
Institution for genotype-based dietary recommendation†,§,‖																	
Family doctor/specialist	373/413	90·3	90·0	91·5	1e–0	97·9	80·1	2e–08	86·9	90·4	98·0	1e–0	100·0	97·1	89·1	93·6	1e–0
Professional nutritional consulting	294/411	71·6	70·8	74·4	1e–0	74·2	68·0	1e–0	72·6	71·2	71·0	1e–0	0·0	86·9	62·4	71·7	2e–03
Health insurance	283/412	68·6	67·3	73·0	1e–0	73·4	61·9	1e–0	65·0	67·2	80·6	1e–0	100·0	75·0	71·8	65·3	1e–0
University/science	302/412	73·3	75·8	64·7	1e–0	74·3	71·6	1e–0	78·0	69·0	80·3	1e–0	100·0	67·0	66·4	83·2	2e–01
Companies/industry	58/411	14·2	15·7	9·0	1e–0	10·6	19·4	1e–0	10·4	16·6	12·9	1e–0	0·0	17·0	14·5	12·7	1e–0
Non-profit organisation	136/407	33·4	34·1	31·2	1e–0	29·8	38·3	1e–0	37·5	29·4	40·0	1e–0	100·0	43·4	25·7	35·3	6e–01

BMI, body mass index; *P*, *P*-value.

Data are statistically weighted by age, gender, education, domicile and BMI.

* Which of the following personal data would you provide for a genotype-based dietary recommendation? Possible answers: yes, no, no answer; shown is ‘yes’ as answer.

† By which institution would you make use of genotype-based dietary recommendations? Possible answers: yes, no, no answer; shown is ‘yes’ as answer.

‡ Persons with answers ‘no answer’ or ‘do not know’ are not included in statistical analysis. The number of those answers can be calculated by the difference between the participants, who got the question (*n* 414), and the number of answers given for each variable.

§ Screening question, which means that only persons who give a certain answer to further item got this question.

‖ After the definition of a genotype-based dietary recommendation was given.

Most of the participants would make use of genotype-based dietary recommendations by their family doctor (90·3 %, 373/413), while 14·2 % (58/411) would make use of it by companies (Table 3). When comparing the percentages between women and men, women would make use of a genotype-based dietary recommendation by their family doctor more often than men (97·9 % *v*. 80·1 %, *P* = 2e–08) (Table 3).

### Setting and effort

Considering the survey participants who would conceive to make use of a genotype-based dietary recommendation (*n* 474), 84 % (83·5 %, 312/374) of them stated that they would make use of a genotype-based dietary recommendation if the concept would be at no charge. Almost 50 % (48·5 %, 191/394) of the participants would also pay for the concept (Table 4). However, the percentage differences showing the willingness to pay for a genotype-based dietary recommendation were statistically significant among the different age subgroups (18–35 years: 55·2 % *v*. 36–65 years: 51·2 % *v*. older than 65 years: 23·7 %, *P* = 2e–02) (Table 4).

**Table 4:** Setting and effort for a genotype-based dietary recommendation (*n* 414)

Variable	Total					BMI (kg/m2)						Gender					Age (years)							Education (years)								
						< 30		≥ 30			*P*	Women		Men		*P*	18–35		36–65		> 65		*P*	Student/none		8/9		10		12/13		*P*
	n/N¶		%			%		%				%		%			%		%		%			%		%		%		%		
**Setting**																																
Usage of free genotype-based dietary recommendation*,**,††	312/374		83·5			82·2		87·4			1e–0	86·6		78·5		1e–0	81·0		84·4		83·3		1e–0	100·0		94·5		82·8		83·2		1e–0
Willingness to pay†,**,††	191/394		48·5			48·2		49·6			1e–0	45·4		52·4		1e–0	55·2		51·2		23·7		2e–02	0·0		55·9		43·6		52·3		1e–0
Approach to genotype-based dietary recommendation‡,**,††																																
One time nutritional consulting via letter or mail	129/352		36·6			38·5		30·9			1e–0	41·7		30·1		1e–0	28·3		37·1		53·3		9e–01	100·0		35·9		25·8		43·1		9e–01
Regularly phone calls for 1 year	85/352		24·2			21·9		31·0			1e–0	23·1		25·5		1e–0	19·5		25·8		28·9		1e–0	0·0		25·0		22·5		21·9		1e–0
Using an application for 1 year	141/352		40·0			40·1		39·8			1e–0	41·7		37·9		1e–0	59·3		34·5		13·3		4e–07	100·0		46·7		33·7		41·3		1e–0
Regularly personal conversations for 1 year	214/352		60·8			56·9		72·5			9e–01	58·3		64·1		1e–0	64·6		60·3		53·3		1e–0	100·0		68·5		66·3		56·3		1e–0
Using an internet platform for 1 year	102/352		29·1			26·3		37·4			1e–0	29·6		28·1		1e–0	31·9		29·9		20·0		1e–0	0·0		27·2		21·3		36·3		1e–0
**Effort**	M				sd	M	sd	M		sd		M	sd	M	sd		M	sd	M	sd	M	sd		M	sd	M	sd	M	sd	M	sd	
Payment (€)§,**,††																																
One time nutritional consulting via letter or mail (*n* 60)	78·3				108·6	84·6	113·1	50·8		85·1	1e–0	68·4	93·2	100·1	136·8	1e–0	92·6	135·0	75·0	103·7	59·6	52·7	1e–0	n. a.		43·4	36·5	89·5	145·7	94·2	118·1	1e–0
Regularly phone calls for 1 year (*n* 50)	182·4				176·8	181·3	133·4	184·5		242·4	1e–0	149·6	135·2	212·9	206·0	1e–0	203·7	217·2	189·5	146·9	97·3	183·2	1e–0	n. a.		79·8	51·4	263·9	282·8	196·4	142·9	1e–0
Using an application for 1 year (*n* 97)	82·2				131·2	76·5	91·1	100·3		221·9	1e–0	78·5	116·1	87·5	155·9	1e–0	77·1	150·4	93·0	119·9	38·0	31·8	1e–0	n. a.		87·2	131·0	104·6	229·2	70·4	83·6	1e–0
Regularly personal conversations for 1 year (*n* 134)	263·6				224·3	273·5	209·7	236·7		261·2	1e–0	225·5	178·1	300·1	257·0	1e–0	309·6	221·4	251·2	219·6	134·8	228·4	1e–0	n. a.		183·7	164·1	347·5	279·0	269·2	208·2	5e–01
Using an internet platform for 1 year (*n* 71)	88·4				113·5	69·4	53·7	132·2		189·4	1e–0	75·4	107·0	105·1	124·6	1e–0	75·0	98·0	101·6	127·7	65·1	102·2	1e–0	n. a.		84·2	155·3	95·2	139·9	86·2	82·3	1e–0
Time effort (min)‖,**,††	49·2				69·9	41·0	60·8	77·2		89·4	4e–04	50·2	74·9	47·7	63·2	1e–0	68·0	83·8	44·3	64·8	31·0	48·4	6e–02	0·0	0·0	42·7	62·0	47·0	66·5	59·9	78·8	1e–0

BMI, body mass index; *P*, *P*-value.

M, mean value; data are statistically weighted by age, gender, education, domicile and BMI.

* If the concept of genotype-based dietary recommendation would be at no charge would you make use of it? Possible answers: yes, no, do not know/undecided, no answer; shown is ‘yes’ as answer.

† Would you pay for the concept of genotype-based dietary recommendation? Possible answers: yes, no, no answer; shown is ‘yes’ as answer.

‡ Which of the following approaches would you prefer to receive genotype-based dietary recommendation? Possible answers: yes, no, no answer; shown is ‘yes’ as answer.

§ How much would you maximal pay for the following approaches inclusively analysing your personal and genotype-based data.

‖ How much time in minutes would you spend regularly per week for maintaining a genotype-based dietary recommendation (*n* 351).

¶ Persons with answers ‘no answer’ or ‘do not know’ are not included in statistical analysis. The number of those answers can be calculated by the difference between the participants, who got the question (*n* 414), and the number of answers given for each variable.

** Screening question, which means that only persons who give a certain answer to further item got this question.

†† After the definition of a genotype-based dietary recommendation was given.

Most (60·8 %, 214/352) of the participants answered that they would prefer regular personal conversations for 1 year (Table 4). Moreover, the participants would pay most for regularly personal conversations for 1 year inclusively analysing their personal and genotype-based data in comparison to the amount of fees for other approaches (Table 4). No statistically significant differences could be found among the different subgroups (Table 4).

The participants would spend almost 1 h per week (49·2 ± 69·9 min) for receiving a genotype-based dietary recommendation (Table 4). The subgroup of persons with a BMI ≥ 30·0 kg/m^2^ would spend about 30 min more time for receiving it than the subgroup of persons with a BMI < 30·0 kg/m^2^ (77·2 ± 89·4 min *v*. 41·0 ± 60·8 min, *P* = 4e–04) (Table 4).

## Discussion

To the best of our knowledge, this is the largest representative survey in Germany analysing the knowledge, opinions and expectations of adults with or without obesity concerning personalised genotype-based dietary recommendations. We have shown that over 30 % of the survey participants indicated a general interest to make usage of genotype-based dietary recommendations. This is in line with two other surveys^(25,26)^. The willingness to participate in genetic risk profiling has been investigated in a non-representative German population of 452 adults. There was an agreement of 45 % of the participants^(25)^. German data from an Europe-wide survey on 5967 persons aged 14 years and older showed that 35 % of the German participants (*n* 991) would do genetic testing for general interest and over 13 % of the German participants would also follow a personalised diet^(26)^. Participants having a poor health status or showing the first symptoms of a disease were more likely to agree with genetic testing^(25,26)^. In the present work, in the subgroup of persons with a BMI ≥ 30·0 kg/m^2^, the willingness to make use of genotype-based dietary recommendations was higher compared to persons with a BMI < 30·0 kg/m^2^. This might be explained by the awareness of being at risk of developing a co-morbidity and of the availability of DTC genetic testing which promise weight loss. In a survey conducted in 2009, 85 % of the participants indicated to do a genetic test for reducing the risk of developing a disease^(27)^. Moreover, the main motivation for undergoing genetic testing was health promotion^(26,27)^.

Besides the BMI-specific differences, several studies have shown that there are also differences concerning gender, age and education and the likelihood of using a genetic test. The present work has shown that no statistically significant difference between women and men and the likelihood of making use of a genotype-based dietary recommendation was found. Other literature has shown that women were more likely to undergo genetic testing than men^(25,26,28)^. Explanations for this might be personal as well as societal issues. For decades, social media displayed the body shape accepted and described diverse forms of diets to achieve it. Therefore, women might be driven by social desirability and personal feelings to make usage of weight-lowering diets more often than men. This is also seen in the present work. Women have tried more often to lose more than 3 kg of their body weight than men. When looking at differences among the age groups in the present work, participants older than 65 years of age were assumed to be less conceived to make use of a genotype-based dietary recommendation than participants aged 65 years and younger. An European-wide survey with 5967 participants indicated a greater willingness for genetic testing of persons older than 65 years of age^(26)^. However, another survey with 1705 participants did not show any significant difference between the age groups below or above 50 years towards the willingness to undergo genetic testing^(29)^. This inconsistency might be explained by the different study designs. As the present work asked for the general conception of making use of genotype-based dietary recommendations, the European-wide survey asked for genetic testing in general and the second survey asked for genetic testing to identify possible predisposition to hypertension. Contrary findings were also seen for the educational impact in using genetic testing. One survey with 1496 chronic patients has shown that participants with higher education were more likely to undergo genetic testing in general^(30)^. In the present work, no significant differences among the education groups and the conception of making use of genotype-based dietary recommendation were seen. However, several studies assumed that the individual knowledge and understanding of genotype-based dietary recommendations is crucial for usage^(30,31)^. In the present work, 19 % of the participants indicated to know the term genotype-based dietary recommendation. In a randomised trial with 149 Canadian adults aged between 20 and 35 years, 30 % of the participants had already heard of a DTC personal genetic test^(32)^. However, a representative survey with 817 adults in the Netherlands could not find any association between knowledge and willingness to genetic testing^(33)^.

As genotype-based dietary recommendations and DTC genetic tests might not be at no charge, the conception of undergoing genetic testing also depends on the willingness to pay. In the present work, almost 50 % of the participants indicated to pay for genotype-based dietary recommendations. Depending on the approach, the participants were willing to pay between 78 to 264 euros. A second European survey that analysed the willingness to pay for personalised nutrition showed that over 80 % of the participants claimed that they would pay for nutritional advice based on lifestyle and genotype^(28)^. However, the German participants of that survey indicated to pay about 54 % of the local reference price (100 euros) including general dietary advice and 3-month follow-up advice^(28)^. This was independent of whether the dietary advice was based on lifestyle, lifestyle and phenotype, or lifestyle and genotype^(28)^. According to focus groups with 126 participants, the price would resemble the quality of a commercially personalised nutritional service^(2)^. A higher price was linked to a higher benefit, better data protection and better qualification of the service provider^(2)^. Moreover, a representative survey in Hungary with 500 adults showed that the higher the attitude towards functional food, the higher the amount of money that would be spend on it^(34)^. However, in the present survey, no BMI-specific difference in the amount of fees for genotype-based dietary recommendations could be found even though the attitude towards genotype-based dietary recommendations was stated to be higher in persons with a BMI ≥ 30·0 kg/m^2^ than persons with a BMI < 30·0 kg/m^2^. This might be explained by a lower socio-economic status of persons with BMI ≥ 30·0 kg/m^2^ than persons with BMI < 30·0 kg/m^2^ resulting in a lower income.

Gender- and education-specific differences in the present survey were also seen when asked for their preferred institution recommending personalised dietary advices. The present work showed that the participants might accept genotype-based dietary recommendations if provided by a medical doctor via regular personal communication. This is in line with other literature^(2,35,36,37)^. A survey in the Netherlands found out that the participants indicated to prefer communication with and delivery of the results through their family doctors^(36)^. Furthermore, a survey demonstrated that personal contact would encourage following genotype-based dietary recommendations and promoting emotional benefits^(2)^. An European survey with 8136 participants found out that personal contact has a positive effect on the privacy calculus which again reduces the privacy risk and therefore increases the personal benefit^(35)^. In addition, the contact with professionals such as the family doctor was preferred, as they are believed to know how to handle genetic testing^(2)^. These results indicate that the medical doctor and personal contacts play an essential role in the usage of a genotype-based dietary recommendation. However, available DTC genetic tests are offered by the industry, which is not the preferred institution by the participants of the present work as well as other surveys^(36,37)^.

Even though most of the participants did not know the term genotype-based dietary recommendation and therefore might not know DTC genetic tests, more than half of the present survey participants believes that genotype-based dietary recommendation is an effective concept in general. Additionally, almost 40 % of the survey participants stated that genotype-based dietary recommendations will become standard in the future. This shows a positive attitude of the German population towards genotype-based dietary recommendations with no differences between analysed subgroups. However, the acceptance of a genotype-based dietary nutrition depends not only on the individual’s attitude but also on the freedom of choice^(38,39)^, the acceptance of family and friends^(38)^ and the knowledge about a personal benefit^(35,38,39)^. Moreover, data protection and therefore the privacy risk are very crucial for genetic testing^(35)^. However, in the present work, over 70 % of the participants would provide material for genotype-based dietary recommendations. This is in line with other literature^(26,40)^. In 1998, willingness to donate blood and to support blood storage for genetic research was named by 43 % of the 2621 participants^(40)^. Stewart-Knox *et al.* described in their Europe-wide survey that the 991 German participants claimed little fear of misuse of the test information by insurers (7·7 %), employers (2·5 %) or authorities or police (6·8 %)^(26)^. This might be explained by the high expectations of a personalised genotype-based dietary recommendation on health, whereby the possible privacy risks are disregarded.

A major strength of the present survey is the representative data. Additionally, experts developed a standardised questionnaire for this project. Moreover, the interviews were standardised based on the computer-assisted telephone-based interviews method and were done by a professional agency. This allowed a high data quality. Furthermore, the data are based on a rather large sample size. By including a large number of participants with a BMI ≥ 30·0 kg/m^2^, results were presented for different BMI classes.

Furthermore, some limitations of the present survey have to be addressed as well. The anthropometric data for the BMI calculation are self-reported. However, several studies have shown that self-report is rather valid for anthropometric data^(41,42)^. We are aware of the fact that the systematic short screening of additional participants with a BMI ≥ 30·0 kg/m^2^ can be criticised form a methodological point of view. However, due to the strict use of a RDD sampling method and statistical weighting for age, gender, education and domicile, we could show representative data for adults in Germany. The subgroup analysis considered only two BMI categories with a cut-off of 30·0 kg/m^2^. As all data are based on self-reporting, we are aware that there might be a self-report bias. As most of the survey participants did not know the term genotype-based dietary recommendations, many answers – despite the presentation of a definition – might be based on imagination and do not represent actual opinions. The discussion of gender-specific differences are the author’s opinions, as based on the questions it cannot be distinguished between social or personal issues. Furthermore, as only thirteen participants were grouped in the student/none education group, the *P*-values must be considered with caution.

## Conclusion

In this survey, knowledge, opinions and expectations of adults concerning personalised genotype-based dietary recommendations were assessed. The results demonstrate that most of the survey participants indicated not to know the term personalised or genotype-based dietary recommendations. Therefore, it is assumed that commercially available DTC genetic tests could not be properly assessed by the general population. However, the survey participants showed interest and willingness towards genotype-based dietary recommendations. Hence, more randomised controlled human intervention trials are needed to investigate genotype-based dietary recommendations for their clinical evidence on weight management.
